# Lack of gut microbiome recovery with spinal cord injury rehabilitation

**DOI:** 10.1080/19490976.2024.2309682

**Published:** 2024-02-07

**Authors:** Ayelet Gur Arie, Itamar Toren, Rotem Hadar, Tzipi Braun, Gilat Efroni, Efrat Glick Saar, Zecharia Madar, Amnon Amir, Gabriel Zeilig, Yael Haberman

**Affiliations:** a Sheba Medical Center, Tel-Hashomer, Tel Aviv University, Tel Aviv, Israel; b Department of Biochemistry, Food Science and Nutrition, The Hebrew University of Jerusalem, Jerusalem, Israel; c Department of Military Medicine, Faculty of Medicine, Hebrew University of Jerusalem, Jerusalem, Israel; d School of health professions, Ono Academic College, Kiryat Ono, Israel; e Faculty of Medicine, Tel Aviv University, Tel Aviv, Israel; f Cincinnati Children’s Hospital Medical Center, Department of Pediatrics, University of Cincinnati College of Medicine, Cincinnati, OH, USA

**Keywords:** Spinal cord injury, gut microbiome, rehabilitation

## Abstract

Spinal cord injury (SCI) is a devastating event that significantly changes daily function and quality of life and is linked to bowel and bladder dysfunction and frequent antibiotic treatment. We aimed to study the composition of the gut microbiome in individuals with SCI during the initial sub-acute rehabilitation process and during the chronic phase of the injury. This study included 100 fecal samples from 63 participants (Median age 40 years, 94% males): 13 cases with SCI in the sub-acute phase with 50 longitudinal samples, 18 cases with chronic SCI, and 32 age and gender-matched controls. We show, using complementary methods, that the time from the injury was a dominant factor linked with gut microbiome composition. Surprisingly, we demonstrated a lack of gut microbial recovery during rehabilitation during the sub-acute phase, with further deviation from the non-SCI control group in the chronic ambulatory SCI group. To generalize the results, we were able to show significant similarity of the signal when comparing to a previous cohort with SCI, to subjects from the American Gut Project who reported low physical activity, and to subjects from another population-based cohort who reported less normal stool consistency. Restoration of the microbiome composition may be another desirable measure for SCI recovery in the future, but further research is needed to test whether such restoration is associated with improved neurological outcomes and quality of life.

## Introduction

Spinal cord injury (SCI) has a global incidence of 15–40 cases per million in high-income countries, with roughly 133,000–226,000 cases annually worldwide,^1^ with traumatic SCI involving predominantly young adult males aged 20 to 29 years. SCI causes various multi-system impairments throughout life, thereby leading to physical and mental disorders,^2^ decreased functionality, and reduced quality of life. Respiratory and cardiovascular conditions, spasticity, pain syndromes,^2^ neurogenic bowel and bladder dysfunction, recurrent hospitalizations,^3,4^ and frequent antibiotic treatment^5^ are common in individuals with SCI. Bowel dysfunction is characterized by autonomic dysreflexia, constipation, incontinence, severe gastrointestinal motor dysfunction, and altered visceral sensitivity.^6^ Severe cases commonly undergo prolonged rehabilitation processes, starting during admission at the rehabilitation unit, known as the subacute phase, and followed by ambulatory clinic rehabilitation at the chronic phase.

Emerging data in human and animal models suggest that gut microbiome composition likely plays a role in intestinal impairment and SCI course.^7^ In mice models, SCI was associated with persistent gut microbial abundance changes, bacterial translocation to visceral organs, and enhanced immune response in gut-associated lymphatic tissue.^8^ It was also suggested that a bidirectional effect on the CNS exists through afferent intestinal vagal feedback,^7^ tryptophan metabolism, endocrine system, and gut microbial metabolites such as short-chain fatty acids.^9^ In humans, previous studies suggest that individuals with SCI had lower microbial diversity,^10,11^ were enriched with unique bacterial taxa,^10–12^ and varied by SCI extent and severity.^5^ Possible confounders to the autonomic-enteric nervous link are the frequent use of antibiotics and dietary changes in SCI patients.^8^ However, few studies have compared the microbiome of SCI subjects between the subacute and chronic phases, and to age- and gender-matched controls, and most lacked longitudinal data and information on whether the gut microbiome composition changes as a factor of time from the SCI accident event and during rehabilitation.

**Figure 1.**
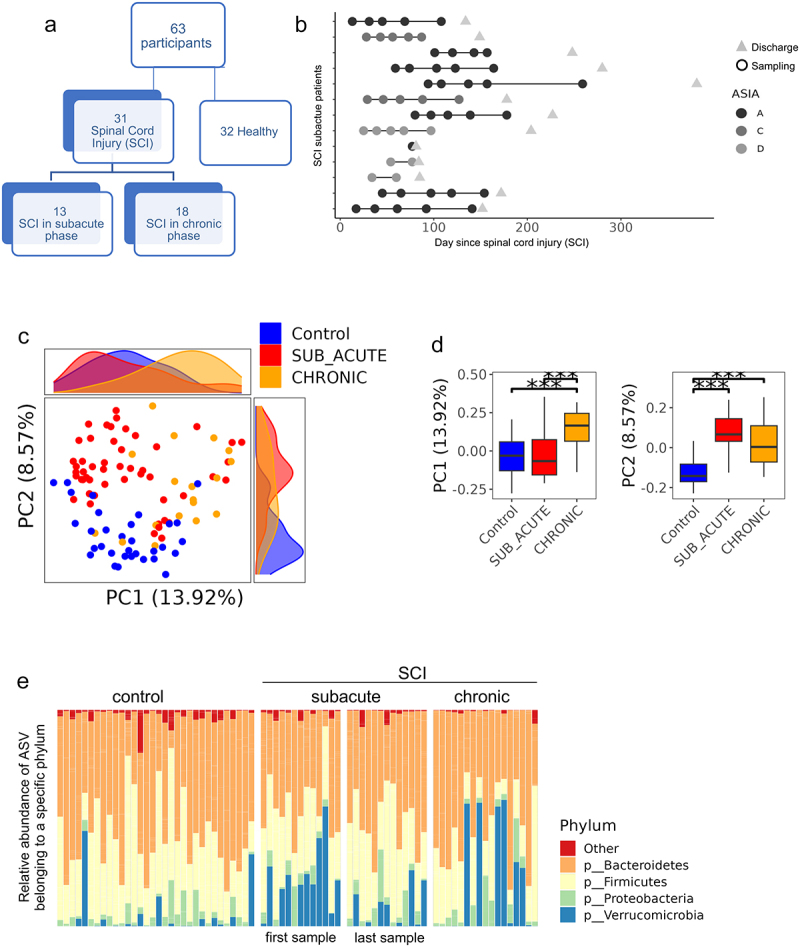
Gut microbiome population differs between controls and SCI sub-acute and chronic patients.

In this study, we set to analyze the short (subacute phase) and long-term (chronic) effects of SCI on the gut microbiome. We aimed to determine if a temporal correlation exists in the rehabilitated SCI patients between time from injury and restoration of the gut microbiome. For this, we included 31 individuals with SCI. Of the 31 subjects, we included 13 patients in the subacute phase who were admitted to the rehabilitation department within a median time from the spinal cord injury of 54 days. This sub-acute SCI group was followed and sampled longitudinally until hospital discharge, which enabled testing dynamics over time shared by SCI subjects and testing specifically if the gut microbiome recovered or deviated from the control signals with rehabilitation and time from the injury. Additionally, we included 18 ambulatory chronic SCI patients who were sampled during their outpatient clinic visits with a median time from the accident of 3588 days, and gender- and age-matched controls without known medical conditions and without SCI (Table 1 and Figure 1A). This analysis highlighted the lack of gut microbial recovery during the rehabilitation process. The SCI-associated signals were compared to other studies showing enrichment for microbes also seen in independent cohorts of patients with SCI, less physical activity, and less normal stool habits.

a. Scheme showing the number and groups of cohort participants. b. Longitudinal sampling (*n* = 50) in the sub-acute SCI group plotted against days from the spinal cord damage or accident. Each row corresponds to a sub-acute SCI patient, with circles representing samples and triangles representing discharge time. Circles are colored by ASIA score. c. Unweighted UniFrac PCoA plot of all 100 16S microbiome fecal samples, colored by group (controls, sub-acute, and chronic SCI patients). Histograms show the distribution of samples and groups against PC1 and PC2 values. d. Boxplots of PC1 (left) and PC2 (right) values from Figure 1C stratified by group. ***p < 0.001, Mann–Whitney test. e. Bar plot of per-sample bacterial relative abundance at the phyla level. Only the top four phyla are shown, and the rest are marked as “Other”. For the longitudinal sub-acute samples, only the first and last samples are shown per patient.

**Table 1.** Cohort demographics and characteristics at study entry.

	Controls (Subject *n* = 32)	SCI sub-acute (Subject *n* = 13, samples *n* = 50)	SCI chronic (Subject *n* = 18)
Age Median (IQR)	39.5 [35.8,44]	45.0 [28,61]	39 [35.3,50.3]
Gender male n (%)	30 (94%)	13 (100%)	16 (88.9%)
BMI Median (IQR)	n = 19 23.5 [22.4,26.3]	n = 13 23.9 [22.8,25.3]	n = 17 23.1 [22.0,25.0]
Smoking n (%)	-	3 (23.1%)	2 (11.1%)
*&Days from spinal cord damage Median (IQR)		54 [28,77]	3588 [741,5622]
Lab available n subject (%)	-	n = 13 (100%)	n = 7(39%)
Hemoglobin Median (IQR)		12.4 [11.7,12.8]	13.8 [12.1,14.3]
#Albumin Median (IQR)		3.2 [3.0,3.6]	3.8 [3.65,3.85]
CRP Median (IQR)		20.2[10.8, 37.1]	19.5 [7.94,46.3]
**ASIA score	-		
A n (%)		8 (62%)	10 (55%)
B n (%) C n (%)		0 (0%) 2 (15%)	4 (22%) 3 (17%)
D n (%)		3 (23%)	1 (6%)
Paraplegia n (%)	-	5 (38%)	9 (50%)
Tetraplegia n (%)		8 (62%)	9 (50%)
^Antibiotics within 1 month of sampling Antibiotics type known (n)	None	10 (77%) n = 10 (100%)	4/17 (24%) n = 4 (100%)
Penicillin		9	0
Cephalosporins		5	2
Fluoroquinolones		6	2
Glycopeptides		1	1
Aminoglycosides		3	0
Nitrofurans		0	1
^^Formula supplementation Complete (Ensure) Protein powder (Easyway)	None	10(77%) 5 5	3 (17%) 2 1

*Days from spinal cord damage to study entry (first sampling).

**ASIA score: A grade is the most severe level of injury, and the impairment is complete with no motor or sensory function below the level of injury. In grade B, the impairment is incomplete. sensory function, but not motor function, is preserved below the neurologic level. In grades C and D, the impairment is incomplete, with different motor functions preserved below the neurologic level.

&*p* value = 0.000633 and #p value = 0.00185 between measurements recorded during first sample in the sub-acute vs. the chronic SCI groups.

^^p < 0.001 between sub-acute vs. the chronic SCI groups.

^reporting only if more than 2 subjects per group. Data extracted from the electronic medical records (EMR).

ASIA; American Spinal Injury Association.

## Methods

### Study population, data, and biospecimens collection

A total of 63 adult participants were enrolled: 31 patients with SCI and 32 controls without SCI. The 31 SCI participants included 13 patients admitted to the rehabilitation department (sub-acute SCI group) and 18 recruited during outpatient clinic visits (chronic SCI group). For the subacute group, participants were all recruited within the first 4 months following injury (median of 54 days, IQR of 28,77) and were all hospitalized as inpatients in the rehabilitation department. For the chronic group, participants were all more than a year from the injury and visited the outpatient clinic. Controls without known disease were age- and gender-matched. The study was conducted in Sheba Medical Center, Israel. Approval from the hospital ethics committee was obtained before commencing this study (No. 5936–19-SMC), and all the participants provided written informed consent. The severity of the injury was defined using the American Spinal Injury Association (ASIA) impairment scale by motor and sensory function preservation.^13^ Clinical data including ASIA grade and level of injury (paraplegia vs. tetraplegia), antibiotic use, dietary supplementation, as well as fresh fecal specimens and blood samples were uniformly collected. Blood tests were drawn per standard protocols and analyzed in Sheba Medical Center’s laboratories for both inpatient and outpatient settings. Laboratory tests included C-reactive proteins (CRP), albumin, and complete blood counts (CBC). Fresh stool samples were aliquoted and frozen immediately at −80C. All patients underwent full physical examinations performed by board-certified rehabilitation physicians, according to the International Standards for Neurological Classification of Spinal Cord Injury (ISNCSCI) standards.^14^ Dietary composition and supplements were evaluated and managed by certified dietitians. Due to the different settings (home and hospital), the SCI groups and controls were not exposed to the same diet. Data regarding enrolled subjects were recorded in a structured manner that included demographic, clinical, and laboratory features. Metadata per sample and subject is detailed in **Dataset S1** including age, gender, time from the injury, and level of the injury.

### DNA extraction, 16S rRNA sequencing, and analyses

Fecal DNA extraction and PCR amplification of the variable region 4 (V4) of the 16S rRNA gene using Illumina adapted universal primers 515F/806 R was conducted using the direct PCR protocol [Extract-N-Amp Plant PCR kit (Sigma-Aldrich, Inc.)].^15–17^ Sequencing was performed on the Illumina MiSeq platform. Reads were processed in a data curation pipeline implemented in QIIME 2 version 2022.2.^18,19^ Reads were demultiplexed according to sample-specific barcodes. Quality control was performed by truncating reads after three consecutive Phred scores lower than 20. Reads with ambiguous base calls or shorter than 150 bp after quality truncation were discarded. Amplicon sequence variants (ASVs) detection was performed using Deblur^20^ resulting in 100 samples with a median of 17,645 reads (IQR 10,586, 29953). ASVs present in less than 1% of the samples were removed resulting in a total of 1337 ASVs. ASV taxonomic classification was assigned using a naive Bayes fitted classifier, trained on the August 2013 Greengenes database, using the QIIME 2 feature-classifier classify-sklearn command. Taxonomy assigned by 16S is indicated by the specific ASV number, the sequence associated with each ASV number is indicated in **Dataset S1** and in the relevant supplementary dataset. All samples were rarefied to 4750 reads for α and β diversity analysis. Faith’s phylogenetic^21^ was used as a measure of within sample α diversity, and Unweighted UniFrac was used as a measure of between sample β-diversity,^22^ using a phylogenetic tree generated by SATé-enabled phylogenetic placement (SEPP).^23^ The resulting distance matrix was used to perform a Principal Coordinates Analysis (PCoA) using the QIIME 2 diversity core-metrics-phylogenetic command. Heatmaps were generated using Calour version 2018.10.1 with default parameters.^24^

Quantifications of variance were calculated using PERMANOVA with the adonis2 function in the R package Vegan,^25^ on the rarefied Unweighted UniFrac distance values. The total variance explained by each variable was calculated while accounting for age, gender and subject as the random variable in the model (except for when looking at the contribution of age and gender, when only age or gender and subject can be controlled for). PERMANOVA was calculated independently for each group and feature. Multivariate Association with Linear Models (MaAsLin2) was used with R package version 1.8.0, to test for specific differentially abundant ASVs between: controls and SCI samples controlling for age and gender and patient ID as the random variable. A false discovery rate (FDR) cutoff of 0.25 was used for all MaAsLin2 analysis.^26^ A new Unweighted UniFrac-based PCoA was generated using only these 169 MaAsLin2 SCI-associated ASVs, using a rarefication depth of 900 reads. PC1 values of this new PCoA were used to summarize SCI-associated bacterial signal.

Comparison to published studies was performed using dbBact^27^ and the dbBact-calour python module. ASV overlap Venn diagrams were created by comparing the set of ASVs positively (i.e. ASVs appearing as “HIGHER IN”) or negatively (i.e. “LOWER IN”) associated with a given dbBact term (e.g. “spinal cord injury”/”physical activity”/”normal stool”) to the sets of ASVs identified in our study, using the dbBact-calour plot_term_venn_all() function. Corresponding p-values were calculated using the Fisher-exact test. In addition, a weighted term f-score analysis was performed by calculating the per-sample frequency weighted sum of the term f-score over all the ASVs present in a given sample, using the dbBact-calour get_term_sample_fscores() function. Briefly, the ASV-term f-score is the harmonic mean of the term precision (i.e., how many of the ASV annotations contain the term) and recall (i.e., how many of the annotations that contain the term contain the ASV). The per-sample term weighted score was calculated by multiplying each ASV f-score by the relative abundance of the ASV in the sample. Between sample comparison was performed using the non-parametric Mann–Whitney test. In the Colombian cohort,^28^ stool consistency was labeled by each local research team within 12 h from stool collection and categorized into 4 discrete levels: “Diarrheic”, “Mushy”, “Normal” and “Hard”. For the comparison with our current SCI cohort, we used only the ASVs significantly differing between the “Normal” and “Hard” groups, using the nonparametric permutation-based rank-mean test with dsFDR multiple hypothesis correction (FDR = 0.1 and dbBact annotation IDs 2918, 2919). For the physical activity-related annotations in the American Gut cohort, we used the self-reported exercise frequency, which is categorized into five discrete levels: “daily”, “regularly (3–5 times/week)”, “occasionally (1–2 times/week)”, “rarely (a few times/month)”, and “never”. For the analysis, we compared the “high exercise” group (comprised of regularly and daily) to the “low exercise” group (comprised of occasionally, rarely, and never). We controlled for confounders by matching the two groups based on age (decade), BMI (underweight/normal/overweight/obese), and gender. A nonparametric permutation-based rank-mean test was used to identify the ASVs differing between the two groups, with a dsFDR multiple-hypothesis correction (FDR = 0.1 and dbBact annotation IDs 1725, 1726). For the American Gut stool consistency-related annotations, we used the self-reported bowel movement quality field, with values: “I tend to have normally formed stool – Type 3 and 4”, “I tend to have diarrhea (watery stool) – Type 5, 6 and 7” and “I tend to be constipated (have difficulty passing stool) – Type 1 and 2”, with numbers corresponding to the Bristol stool score. “hard stool” and “normal stool” annotations were added by comparing the “I tend to have normally formed stool – Type 3 and 4” and “I tend to be constipated (have difficulty passing stool) – Type 1 and 2” groups. We controlled for confounders by matching the two groups based on age (decade), BMI (underweight/normal/overweight/obese), and gender. A nonparametric permutation-based rank-mean test was used to identify the ASVs differing between the two groups, with a dsFDR multiple hypothesis correction (FDR = 0.1 and dbBact annotation IDs 8447, 8448).

For Predicting bacterial KEGG ontology enrichment, we used a previously used approach^29^ with several modifications. Briefly, for each of the ASVs identified using MaAsLin2 as significantly associated with SCI or controls (as described above) the KEGG ontology gene functions were predicted using picrust2 intermediate tables as produced by picrust2_pipeline version 2.5.0.^30^ For each ASV, KEGG ontology terms were then aggregated at KEGG level 3 and converted to per-ASV relative level 3 abundance. ASVs were further aggregated at the genus level (aggregating the predicted level 3 functions over all ASVs from the same genus) and transformed to binary (i.e. presence/absence of the level 3 KEGG ontology in the genus aggregated ASVs). A non-parametric, permutation-based rank-mean differential abundance test with multiple hypothesis correction (dsFDR = 0.1) was then used to identify the level 3 KEGG ontology terms enriched in the group of ASVs higher in controls or SCI patients (as identified using MaAsLin, i.e. level 3 KEGG ontology functions present significantly more in the group of ASVs higher in controls or SCI patients).

### Statistical analysis

Statistics were performed in R with the details mentioned above. Overall, Pearson’s chi-square test or Fisher’s exact test was used for categorical variables, Spearman’s rank correlation was used for continuous variables, and the Mann-Whitney U-test for categorical variables, with Benjamini-Hochberg Procedure for FDR correction.

### Data availability

The 16S amplicon sequencing dataset was deposited in the National Center for Biotechnology Information as BioProject PRJNA1007383.

## Results

### Characterizing gut microbiome composition in different rehabilitation stages after SCI

We collected fecal samples and metadata from individuals with SCI and from controls without known medical conditions and without SCI (Table 1 and Figure 1). The 31 SCI participants (94% males) included 13 patients admitted to the rehabilitation department and recruited to the study within a median time of 54 days [interquartile range (IQR) 28–80 days] from the onset of the spinal damage defined as the sub-acute SCI group. Subjects in the sub-acute group were followed and sampled longitudinally about every month until hospital discharge, for a total of 55 longitudinal samples (Figure 1B). Additionally, we included 18 ambulatory chronic SCI patients that were sampled during their outpatient clinic visits with a median time from the onset of the injury of 3588 days (IQR 741–5622 days). This group was seen in the clinical setting. Age, gender, and BMI did not differ between the groups. All participants were consuming a non-restricted diet, and there were no vegetarians or vegans. Antibiotics were frequently used in SCI participants, and formula supplementation was significantly higher among the sub-acute SCI vs chronic SCI group (10/13 vs 3 of 18, chai square *p* < 0.001). All SCI subjects suffer from constipation and require medication used for constipation (stimulants and laxatives), and enemas for defecation.

The International Standards for Neurological Classification of Spinal Cord Injury (ISNCSCI)^14^ represents the gold standard assessment for documentation of the level and severity of a spinal cord injury (SCI). The severity of the injury is defined by the American Spinal Injury Association (ASIA) impairment scale by motor and sensory function preservation.^13^ Rehabilitated patients were classified by SCI ASIA severity score; 61.5% of the sub-acute had complete (A grade) lesion which remained unchanged until discharge and similarly 55.6% of the chronic group had an A grade lesion. In addition, 32 age- and gender-matched subjects without SCI were included, serving as a control group (Table 1 and Figure 1). When comparing the available lab results of the chronic SCI group to the first available lab results in the sub-acute group, the sub-acute group showed reduced albumin levels, with no differences in hemoglobin and CRP levels (Table 1).

### SCI gut microbial population differs from controls, with more substantial differences noted in the chronic SCI group

Microbial composition was determined using the V4 16S rRNA amplicon sequencing characterizing amplicon sequencing variants (ASVs) relative abundance. Principal Coordinates Analysis (PCoA) was used to visualize the variation and similarities between samples’ microbial composition (Figure 1C). PCoA analysis shows distinct clustering of the samples into the three main groups – controls, sub-acute and chronic patients (PERMANOVA *p* = 0.001, 999 permutations), with the chronic patient having significantly different PC1 values (Mann – Whitney test *p* < 0.001), and controls having significantly different PC2 values (Mann – Whitney test *p* < 0.001) from the other two groups (Figure 1D). To avoid a within-patient bias, the longitudinal sub-acute samples were stratified to include only the first or last sample per patient, showing similar PCoA results (Figure S1, PERMANOVA *p* = 0.001 for both first and last sample per patient, with 999 permutations).

**Figure 2.**
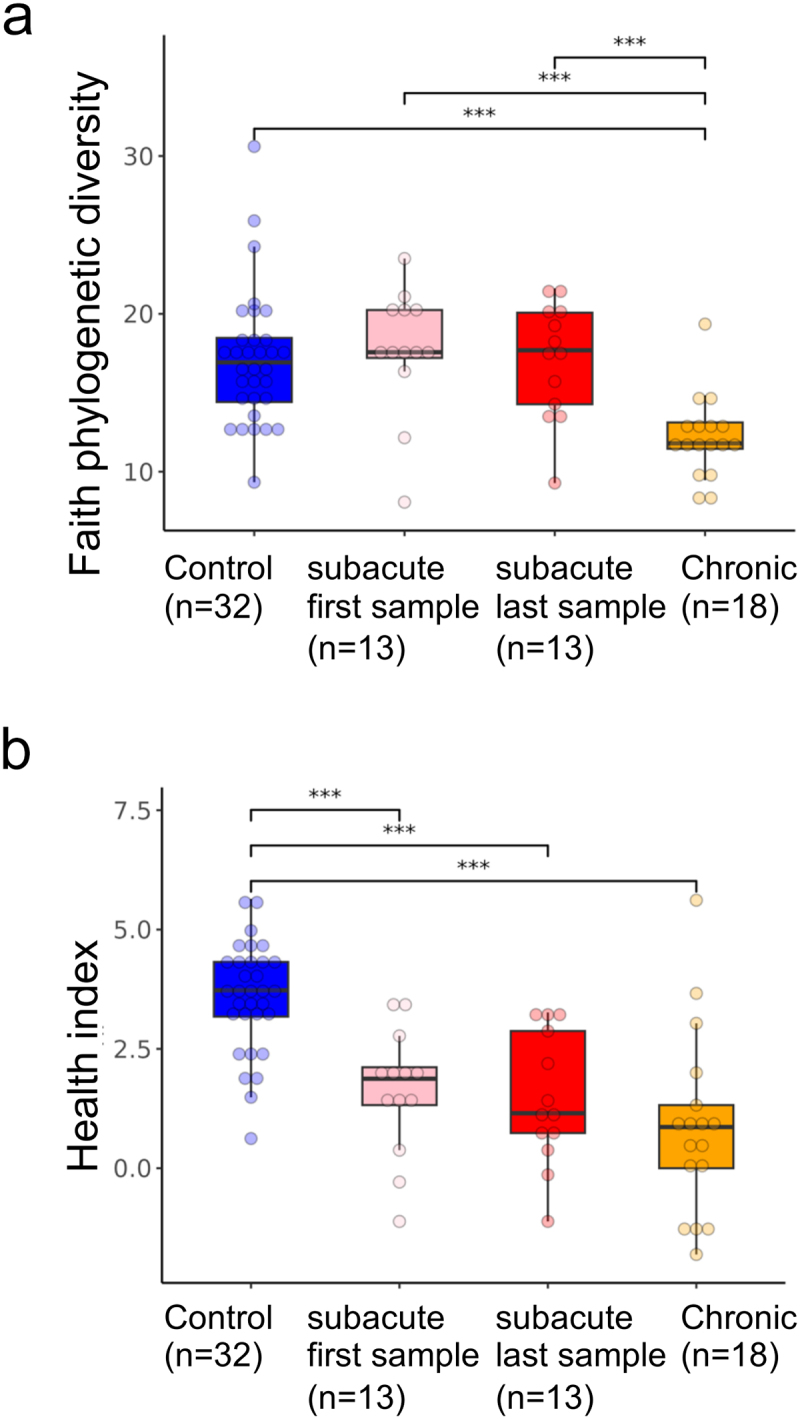
Alpha diversity and health index changes in SCI.

The relative abundance bar plot at the phyla level shows significantly reduced levels of Firmicutes in the chronic patients compared to controls and sub-acute last sample (Mann – Whitney test *p* < 0.009), with a similar trend seen when comparing chronic vs. first sub-acute sample (Mann – Whitney test *p* = 0.056). *Verrucomicrobia* was higher in the sub-acute first sample compared to controls (Mann – Whitney test *p* = 0.00074, Figure 1E). Faith-based Alpha-diversity values were reduced in chronic patients compared to sub-acute and controls (Figure 2A, Mann – Whitney test *p* < 0.001). Previous studies have shown a common gut microbial signature shared across multiple diseases.^29,31^ We therefore applied a previously defined microbial health index^29^ to characterize the level of disease vs. health-associated bacteria. This health index was reduced in both sub-acute and chronic SCI patients relative to controls (Figure 2B, Mann–Whitney test *p* < 0.001), indicating a more disease-like state in SCI patients. Overall, SCI patients exhibit distinct microbial changes compared to controls, with the sub-acute group being in between controls and chronic patients. Sub-acute SCI patients were more similar to controls in their PCoA PC1, alpha diversity, and Firmicutes values, while more similar to chronic SCI patients in their PCoA PC2 and health index values. To test for the effect of antibiotics, we stratified the groups by antibiotics exposure within 4 weeks and included one sample per subject (first or last sampling in the subacute SCI group, Figure S2). This still indicated a significant reduction in Faith-based alpha diversity in the SCI chronic group, with a significantly higher health index in controls, with a trend for higher *Verrucomicrobia* between SCI first and control and last subacute samples.

a. Boxplot of alpha diversity values, using Faith’s phylogenetic diversity, between control, sub-acute, and chronic SCI patients. Only the first and last samples were used for the longitudinal sub-acute samples, to avoid a within-patient bias. b. Boxplot of our previously defined health index^29^ between control, sub-acute, and chronic SCI patients. Again, only the first and last samples were used for the longitudinal sub-acute samples, to avoid a within-patient bias. ***p < 0.001, Mann–Whitney test. Total n is shown in brackets.

### Time from the spinal cord damage is a dominant factor affecting the gut microbiome

**Figure 3.**
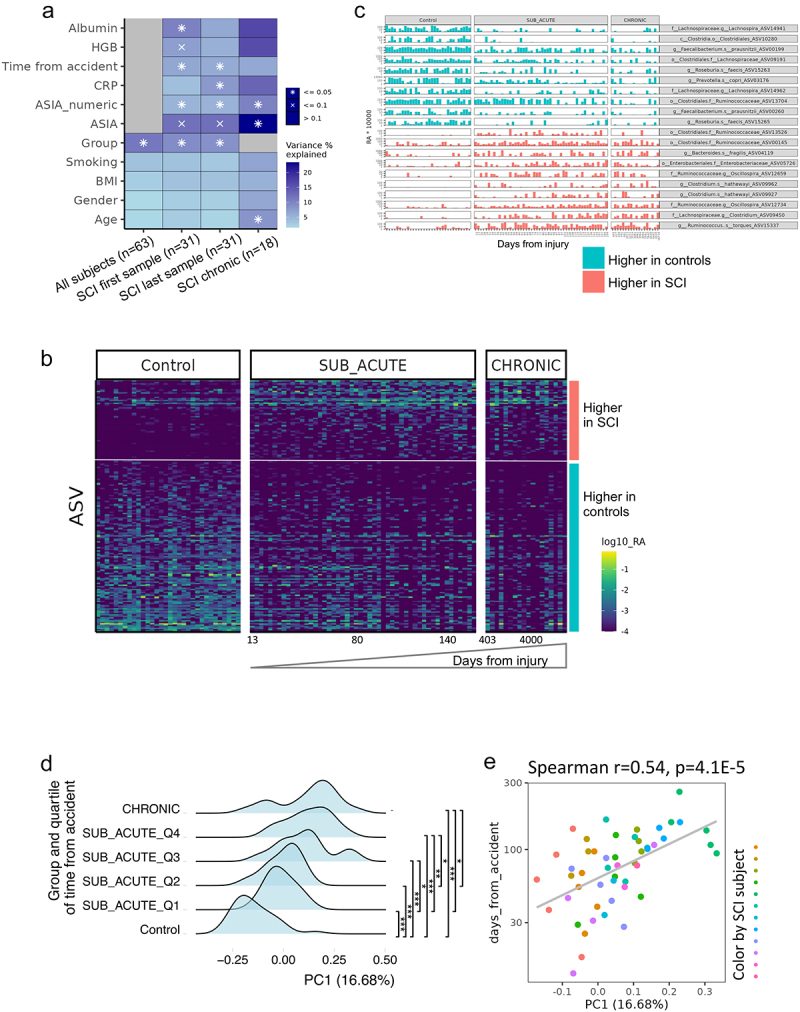
Time from spinal cord damage influences the gut microbiome.

Permutational multivariate ANOVA (PERMANOVA) was used to evaluate the contribution of different factors to overall gut microbiome variation. PERMANOVA was run using only one sample per subject, to control for subjects’ contribution, while controlling for age and gender. PERMANOVA was run separately on all participants (SCI and controls *n* = 63, first sample at study entry), with only SCI subjects (*n* = 31) but including the first samples of the sub-acute group close to admission at the rehabilitation department, and with one sample per SCI subject (*n* = 31) including the last sample of the sub-acute group prior to discharge (Figure 3A, see Methods). The subject’s group (control, sub-acute or chronic) consistently explained a high percentage of the microbial variation (8–11%), and time from spinal cord damage and accident accounted for ~6% of the variance within patients. Interestingly, ASIA score, CRP, HGB, and albumin were all significantly associated with microbial variance to a certain extent, while gender, age BMI, and smoking were not significant (Table S2).

a. PERMANOVA analysis of microbial variation explained by metadata, run separately for the first sample of all subjects, and for the first and last sample within SCI patients, to avoid a within-subject bias. Statistical significance is marked. Variance is estimated for each feature independently while accounting for age and gender (see Methods section). Total n is shown in brackets. b. Heatmap showing the 169 ASVs significantly different between controls and SCI patients, while controlling for age, gender, and subject, using all 100 samples. Each row represents an ASV and each column a sample. Samples are stratified by group and ordered by time from the accident, showing a shift in the longitudinal sub-acute samples into a state more similar to chronic patients. c. Bar plot showing the top 10 increasing and top 10 decreasing ASVs between controls and SCI patients from B. Samples are stratified by group and ordered by days from injury. Relative abundance (RA) values were multiplied by 10,000 to enable the use of a log scale. d. Ridgeline plot showing the distribution of SCI-associated PC1 values between the different groups. PC1 values were extracted from the Unweighted UniFrac-based PCoA calculated on the SCI-associated 169 ASVs from Figure 3B. Groups include control, chronic SCI, and sub-acute SCI, where the longitudinal sub-acute SCI group samples were stratified into four quartiles by time since the accident. *q < 0.05, **q < 0.01, ***q < 0.001, Mann–Whitney test with Benjamini – Hochberg FDR correction. E. Scatter plot of the same PC1 values from Figure 3C against the number of days since the accident, within the 50 longitudinal sub-acute SCI samples, colored by subject, with a linear regression fitting.

**Figure 4.**
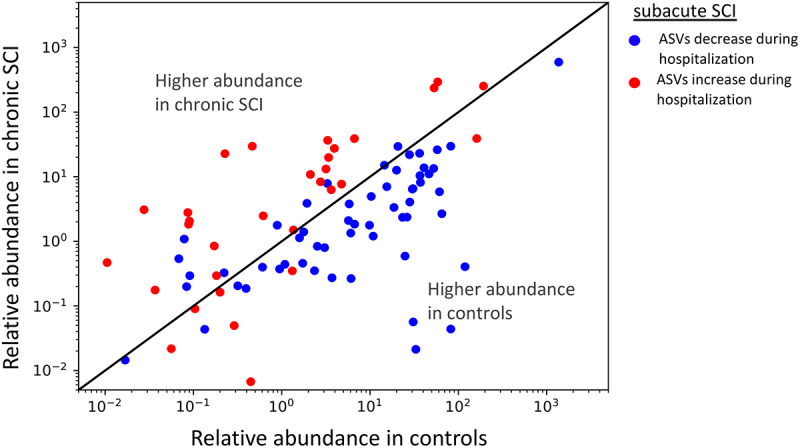
Longer time from the spinal cord damage in the sub-acute group linked with bacteria increased in chronic SCI.

To identify specific microbial ASVs associated with SCI compared to controls, we used Multivariate Association with Linear Models (MaAsLin). The analysis was controlled for age, gender, and subject. One hundred and fifteen ASVs were significantly increased in controls compared to SCI patients, including *Faecalibacterium prausnitzii and Prevotella copri*. Fifty-four ASVs were increased in SCI patients, including *Ruminococcus gnavus* (Figure 3B, Table S3, p < 0.05, FDR < 0.25). Interestingly, when looking at the ASVs associated with overall SCI by time since the spinal cord damage, it seems there is a gradual shift in the longitudinal sub-acute samples, that start from a more control-like state to a state more similar to the chronic SCI patients (Figure 3B–C). To verify and quantify this observation, we calculated the PCoA PC1 values of all samples using only the MaAsLin SCI associated ASVs from Figure 3B. A histogram of these PC1 values, where the longitudinal sub-acute SCI samples were stratified into four quartiles by time since the accident, demonstrates a similar shift from control to chronic state (Figure 3D). Control samples PC1 values were significantly different from all SCI subgroups stratified by time from injury (Mann – Whitney test with Benjamini-Hochberg FDR correction q < 0.001), while chronic SCI samples were only significantly different from the first and second quartiles sub-acute sample (Mann – Whitney test with Benjamini-Hochberg FDR correction q < 0.05). Additionally, within the sub-acute samples, time from the spinal cord damage and accident was correlated with SCI-associated PC1 values (Figure 3E, Spearman’s *r* = 0.54, *p* = 4.1E–5). To supplement these analyses, we also characterized the bacterial ASVs that are altered in the sub-acute SCI group as a factor of time from the accident. We identified 62 and 83 ASVs positively and negatively correlated with time from the accident (Spearman correlation, FDR = 0,25). For each of these ASVs, we plot the mean abundance in the healthy control group and the chronic SCI group (x- and y-axis in Figure 4 respectively). The majority of the ASVs that decreased in the sub-acute group during hospitalization and as a factor of the elapsed time from the spinal cord damage are higher in the healthy control group compared to the chronic SCI group. Conversely, ASVs that increased during hospitalization in the sub-acute group, are also higher in chronic SCI compared to the healthy controls (two-sided binomial test p-value <4E–8). As the control and chronic SCI groups are independent of the sub-acute SCI group, this indicates that the microbial change occurring during sub-acute SCI is not resolved during the long rehabilitation in the chronic SCI group. To further identify gene functions differing between healthy and SCI, we performed a picrust2-based analysis of predicted enriched functions in ASVs associated with the healthy and SCI groups (Figure S3 and dataset S3). We observed a significant enrichment of several pathogenicity-associated KEGG ontology level 3 groups in the SCI-associated ASVs including antimicrobial resistance genes (FDR = 0.037), biofilm formation (FDR = 0.032), and Bacterial invasion of epithelial cells (FDR = 0.04). Only a single statistically significant KEGG ontology level 3 group was significantly enriched in the healthy-associated ASVs, and it was the secondary bile acid biosynthesis (Figure S3, FDR = 0.08).

X and Y axes represent the mean relative abundances of each ASV in the chronic and control groups (above and below the slope line respectively). Shown are ASVs significantly positively and negatively correlated to time from injury in the sub-acute patient group (blue and red colors respectively), using Spearman correlation (FDR = 0.25). Most of the ASVs increasing during the sub-acute SCI phase are also higher in the chronic group compared to healthy controls, whilst ASVs decreasing in the sub-acute phase are higher in healthy controls over the chronic SCI group (above and below the slope respectively, two-sided binomial test p-value <4e-8).

### Comparison of the microbial signal shows some similarities with another cohort with SCI, less physical activity, and hard stool consistency

**Figure 5.**
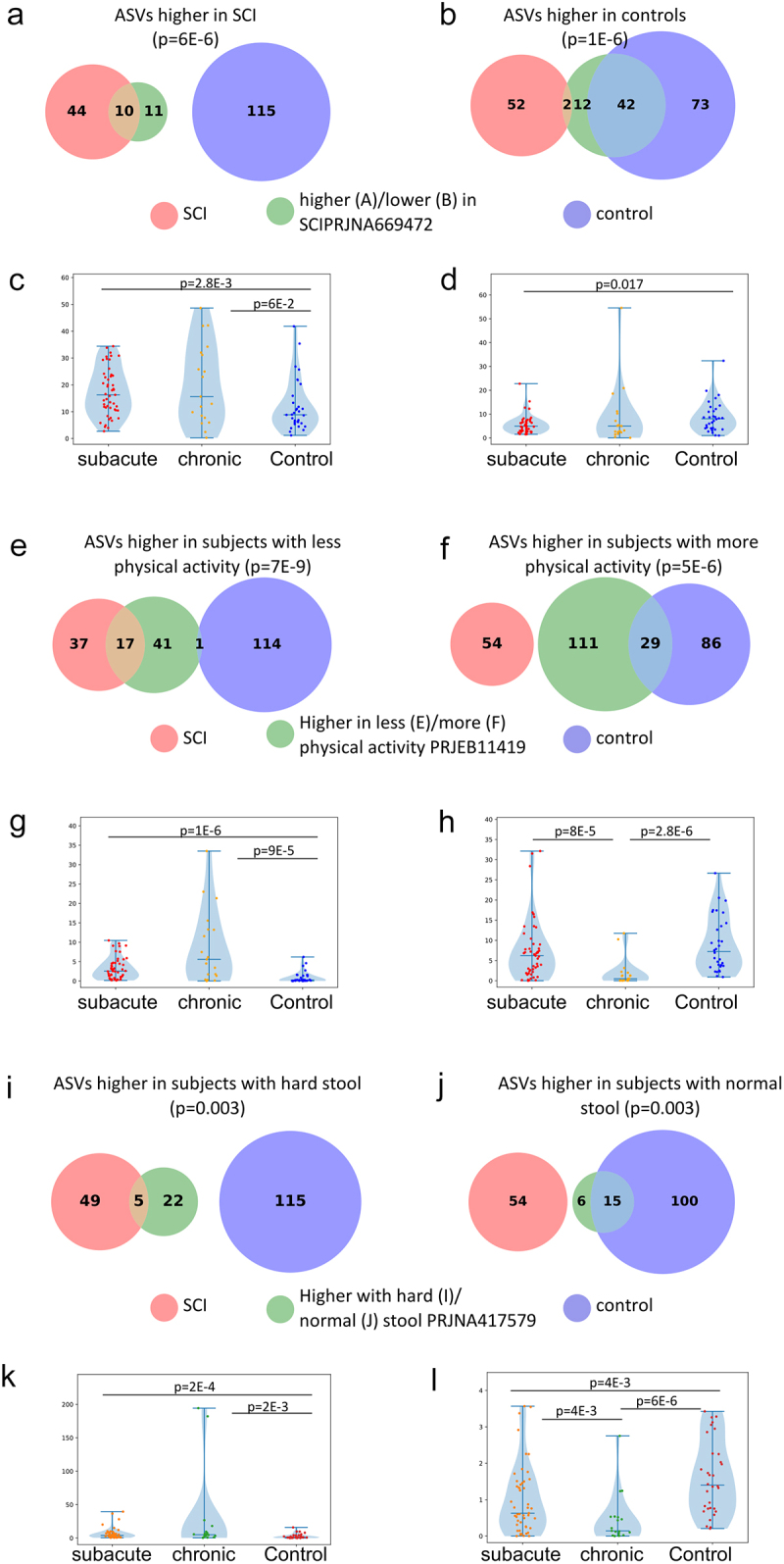
Similarities between bacteria associated with SCI, physical activity and stool consistency.

In order to validate the generality of our results, we compared the SCI-dependent ASVs identified in our cohort (i.e., the set of 54 and 115 ASVs that were higher and lower respectively between SCI and controls after controlling for age, gender, and BMI using Maaslin, Table S3) to previous datasets. To prevent the effect of study–study differences, in each external dataset, we first performed an analysis to identify the associated ASVs within the external study, and then compared this set of ASVs from the external study to the SCI-associated ASVs identified within our current study (see Methods section for details). We first compared our results to the results of a Chinese SCI cohort^32^ including 21 complete thoracic SCI (2–12 months post-injury) and 24 healthy controls annotated in dbBact.^27^ Of the 21 ASVs that are identified as higher in SCI compared to controls in the Chinese cohort, 10 (out of 54) are also higher in SCI compared to our cohort, whereas 0 (out of 115) are higher in controls compared to SCI in our cohort (Figure 5A), showing a significant agreement in directionality (Fisher exact p-value = 6E–6). Of the 56 ASVs higher in controls in the Chinese SCI cohort, only 2 ASVs (out of 54) and 42 (out of 115) overlapped with ASVs lower and higher in SCI and controls in our cohort respectively (Figure 5B, Fisher exact p-value = 1E–6), showing a significant agreement in directionality. As this analysis is based on the presence of a specific ASV, we also performed complementary analysis by calculating for each sample in our cohort, the frequency-weighted dbBact term f-score, which represents the term enrichment by ASV abundance per sample of the previously published cohort in our groups (sub-acute SCI, chronic SCI, and controls), where a higher score indicated higher enrichment. Based on the Chinese cohort higher/lower in SCI annotations, the per-sample f-score for “spinal cord injury” is significantly lower in our healthy control group compared to the sub-acute and chronic groups (Figure 5C, Mann–Whitney p-values 0.06, 0.003 for the control vs. chronic and control vs. sub-acute respectively). A similar analysis for “lower in spinal cord injury” shows higher f-scores for the control group compared to the sub-acute and chronic groups (Figure 5D, Mann–Whitney p-value = 0.017 for the control vs. sub-acute group).

a. Overlap between ASVs significantly higher in SCI in our study (red circle), ASVs significantly higher in healthy controls (blue circle), and ASVs significantly higher in SCI compared to controls in a previously published Chinese cohort^32^ (green circle, dbBact annotation ID 7428), showing similar SCI disease signals (Fisher exact’s p-value <6e-6). b. Similarly to 5A (red and blue circles remained as 5A) but comparing to ASVs significantly lower in SCI compared to controls in the mentioned Chinese cohort (green circle, dbBact annotation ID 7427), showing consistent healthy control signal (Fisher exact’s p-value <1e-6). c. Per sample distribution of the weighted dbBact^27^ F-score for the term “higher in spinal cord injury” across the three cohort sub-groups (Distribution lines representing min median and max for each subgroup) showing that the bacterial composition of healthy controls from our study are less associated with SCI bacteria identified in the previous Chinese cohort^32^ (Horizontal bars denote significant differences between groups with mentioned p-values by nonparametric Mann-Whitney tests). d. Similarly to 5C, but comparing F-score for the term “lower in spinal cord injury”. The bacterial composition of healthy controls from our study is more associated with control bacteria identified in the previous Chinese cohort^32^ e-h. Similar to 5A-D but comparing to bacteria positively/negatively associated with self-reported physical activity in the American Gut Project^33^(dbBact annotation IDs 1726 and 1725 respectively), showing microbial associations between SCI to lower self-reported physical activity. i-l. Similar to 5A-D but comparing to bacteria positively/negatively associated with normal/hard stool consistency in the Colombian population-based cohort of over-weight adults^28^, (dbBact annotation IDs 2918 and 2919 respectively), showing microbial associations between SCI and hard stools.

A similar analytic approach was applied to compare the SCI-dependent ASVs from our cohort (Table S3) to additional dbBact studies to identify connections between these ASVs and other factors affecting the gut microbiome. We performed term enrichment analysis using dbBact,^27^ in order to shed light on unique disease signals. After deduction of general health terms, we focused on two terms potentially related to the SCI disease course: physical activity level, using the American gut cohort,^33^ and hard stool consistency, using a Columbian population-based study.^28^ These terms were selected as SCI subjects experience disability and all use medication used to treat constipation. ASVs higher in SCI in our cohort showed significant similarity to ASVs observed in individuals with low physical activity (Figure 5E–G), whereas ASVs higher in controls in our study showed significant similarity to ASVs observed in individuals with high physical activity (Figure 5F–H). When examining the relation with stool consistency, ASVs higher in SCI in our cohort were significantly similar to ASVs associated with hard stool (Figure 5I–K), whereas ASVs higher in controls in our cohort were significantly associated with normal stool (Figure 5J–L). The similarity of our SCI-associated ASVs with hard stool-associated ASVs was further replicated in an additional analysis regarding stool consistency using the American Gut cohort (Figure S4). Results are similar to the Colombian cohort results, with healthy controls sharing a large fraction of the normal stool-associated ASVs, and SCI patients similar to the constipated associated ASVs.

## Discussion

Rehabilitation post-SCI is a complicated process, and investigations into its shortcomings could lead to innovations promoting quality of life and longevity.^34^ Using patient data-driven approaches, we demonstrated a lack of gut microbial recovery over time in both sub-acute and chronic individuals with SCI. Using complementary methods and a subset of SCI subjects with longitudinal sampling, we demonstrate that time from the spinal cord damage is a dominant factor affecting the gut microbiome, with a lack of recovery during rehabilitation in the sub-acute group, and a more severe deviation from controls detected in the chronic phase. Although it is well recognized that the rehabilitation process positively affects many biological systems,^34^ we demonstrate a lack of gut microbial recovery over time demonstrated by altered microbial patterns, reduced bacterial diversity, and lower universal health index.^21,29^ Comparing our results with previous publications,^28,32,33^ we captured similarities in the microbial signal detected in a previously reported SCI study. Interestingly, this signal was also enriched in subjects performing lower physical activity and in those considered to have hard stool, relevant in subjects with neurogenic bowel dysfunction^6,35,36^ seen with SCI. Recovery of the microbiome can be another desirable measurement of the future SCI rehabilitation process, which can potentially be achieved with more diverse dietary exposure, more activity, and less use of antibiotics. However, more work is needed to support such a concept and specific interventional studies are needed to support such a concept.

Alteration in the gut microbiota post-SCI could also be related to reduced patients’ physical mobility and gut motility or related to comorbidities such as intestinal functional disease, mood and anxiety disorders, susceptibility to infections, altered lipid metabolism, chronic pain,^9,37^ and frequent antibiotics use. All these factors are part of the overall conditions, and it is difficult to separate them apart in our cohort. However, to account for some of the variance attributed to the systemic conditions, we captured hemoglobin and albumin blood levels (available for a subset), previously shown to correlate with both neurological and non-neurological outcomes in SCI patients,^38,39^ which were either significantly lower (albumin) or showed a nonsignificant downward trend (hemoglobin) in the sub-acute group comparing to the chronic group (Table 1), highlighting that the more severe effect on the gut microbial population, as a factor of time from the spinal cord damage, is not correlated with these factors. Systemic inflammation and increased CRP were previously linked with leaky gut biomarkers,^40^ and may interfere with the SCI rehabilitation process.^7,41^ However, in our cohort for the available subjects, there was a non-significant difference in CRP between the chronic and sub-acute SCI groups.

Our study has several strengths and some limitations. Our cohorts included subjects in the sub-acute phase with longitudinal follow-up and subjects in the chronic SCI phase. Using longitudinal sampling in the subacute group enabled capturing microbial community changes over time in response to the injury. We demonstrated striking unexpected results showing a lack of gut microbial recovery with rehabilitation. We further validated the SCI signal with other previously published SCI cohorts. Stool and blood specimens were analyzed in a single center minimizing laboratory differences in protocol and methodology. Limitations consisted of a relatively small sample size, with limited data regarding dietary preferences, physical activity, bowel habits, and other unknown confounders. We note that SCI is not a prevalent condition and often SCI patients suffer from other debilitated diseases, thus limiting the recruitment. Possible coexisting confounders include dietary alterations, reduced mobility, and the fact that patients in the SCI were exposed to recurrent antibiotic use. While these and additional confounders likely contribute to the net effect, they are part of the post-injury new reality of the SCI population, and likely contribute to the lack of recovery and worsening deviation from healthy microbial composition seen in SCI patients. Further evaluation in additional larger cohorts may enable the validation of our results and estimation of the contribution of these coexisting confounders. Finally, our microbial analysis included 16S amplicon sequencing with picrust2 prediction for bacterial function enrichment. While such predictions have been shown to perform well in human gut microbial populations, direct measurement of bacterial functions via shotgun metagenomic sequencing can provide more accurate results.

In summary, we showed a lack of gut microbial recovery during the SCI rehabilitation process in the sub-acute phase during hospitalization, which is worsened in the group with chronic SCI with long-term ambulatory clinic follow-up. Recovery of the microbiome can be another desirable measurement of future SCI rehabilitation, but more work is needed to support such a concept and test if it improves SCI outcomes and quality of life.

## Supplementary Material

Supplemental Material
