# Supplementary material for: Lack of gut microbiome recovery with spinal cord injury rehabilitation
Source: Gut Microbes. 2024 Feb 7;16(1):2309682. doi: 10.1080/19490976.2024.2309682 (PMC10854366; doi:10.1080/19490976.2024.2309682)
Supplement: Supplemental Material [file KGMI_A_2309682_SM3331.zip › SCI_suppl_Figures_Nov23_2023.docx]

#
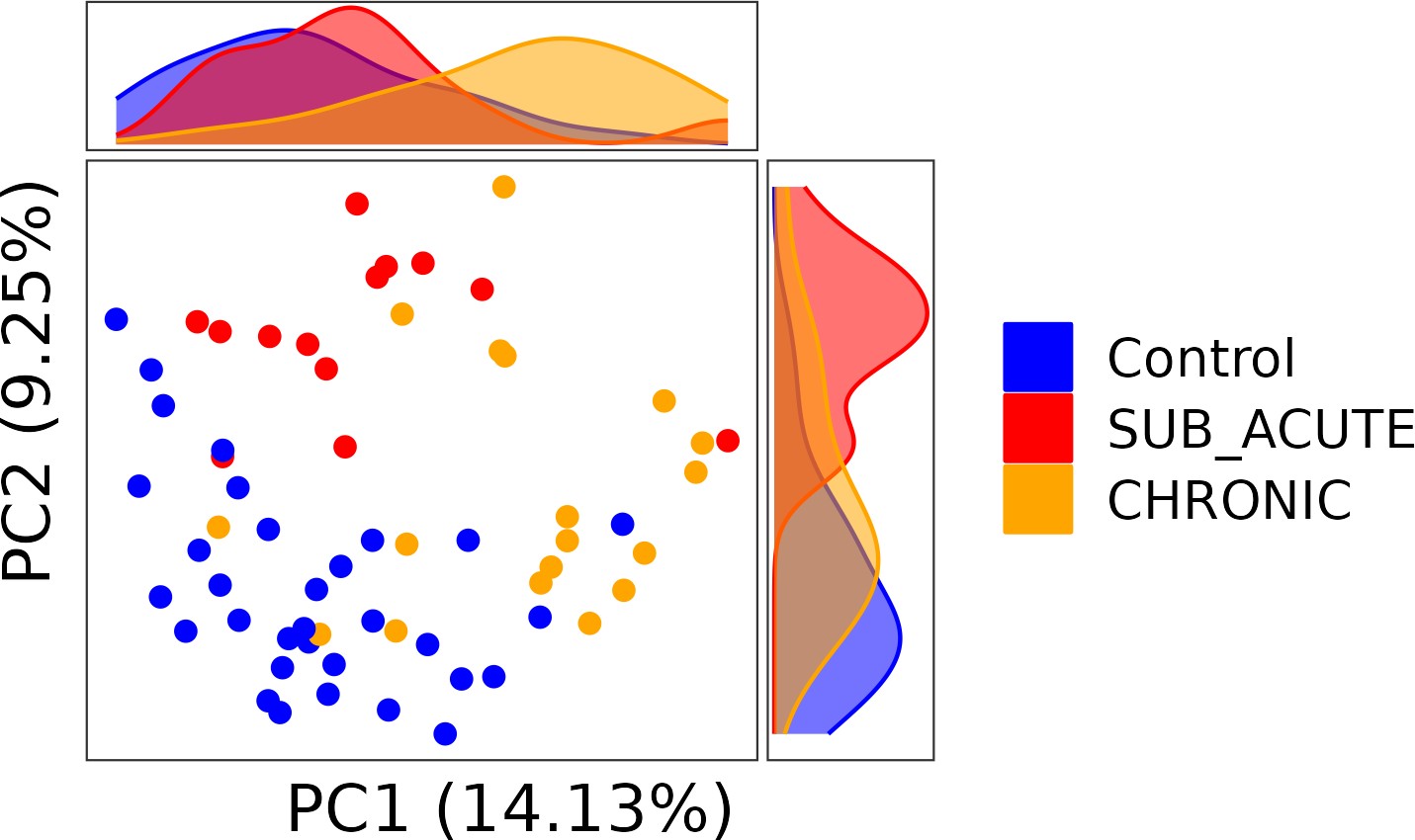
First sample


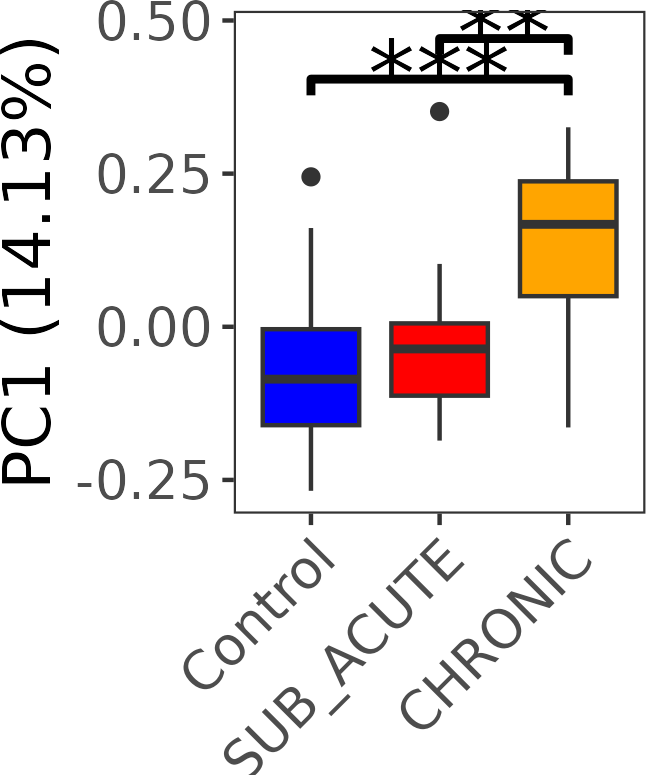

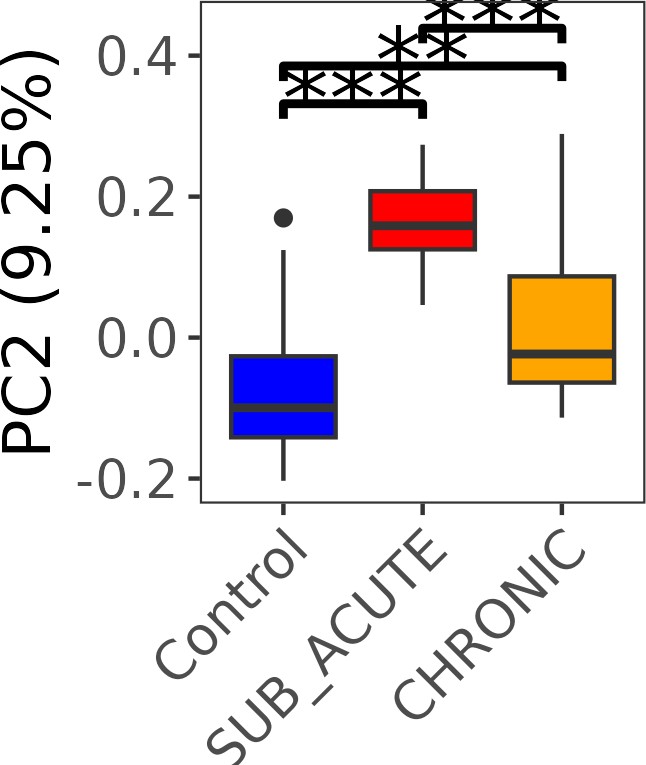


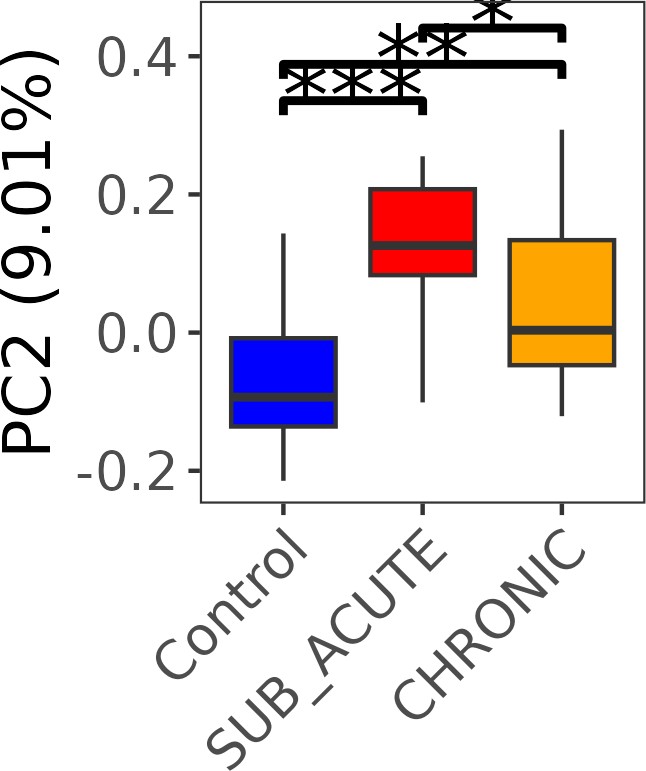


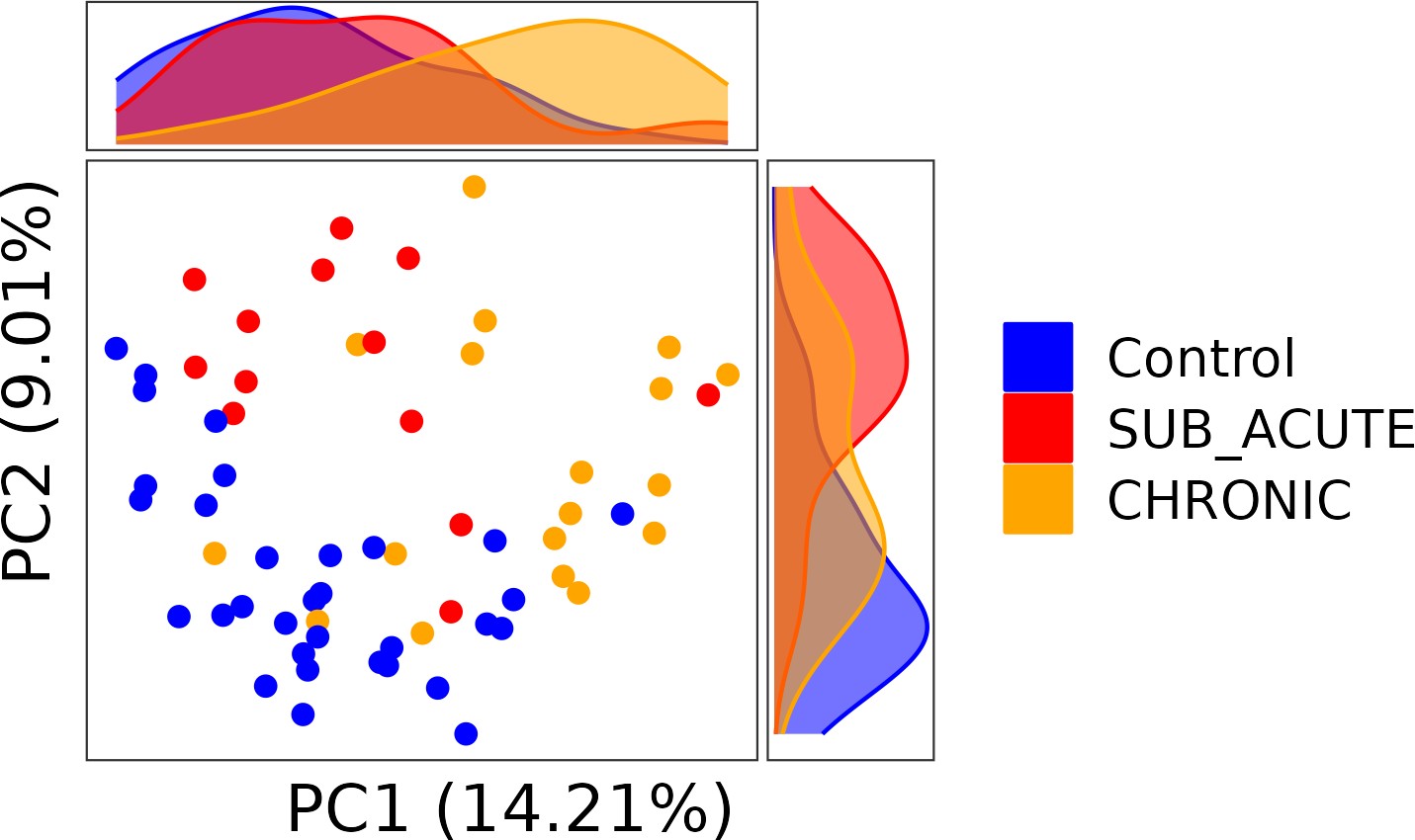

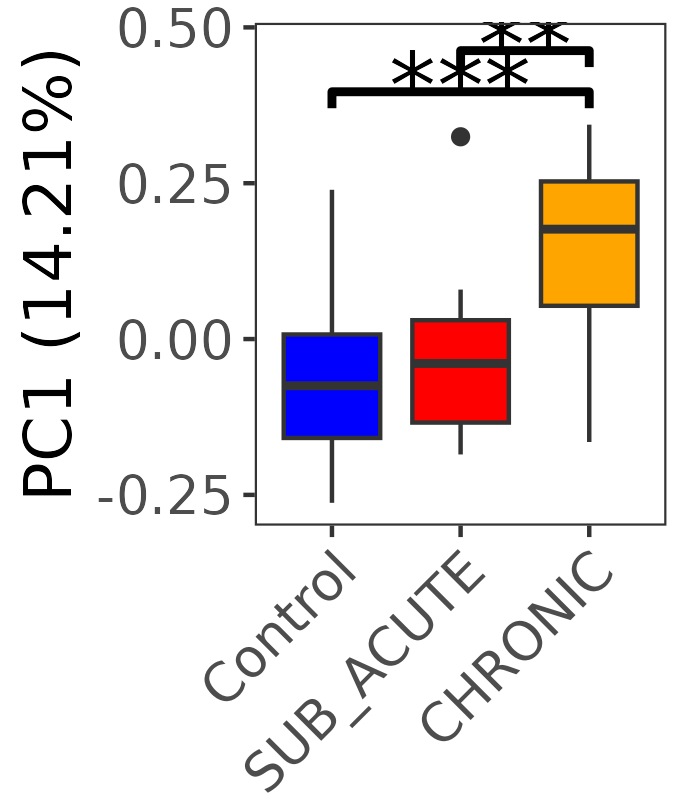


Last sample

**Fig. S1. Unweighted UniFrac PCoA plot of 16S microbiome fecal samples colored by group** (controls, sub-acute, and chronic SCI patients). This figure is similar to Fig. 1C, using only one sample per patient, to avoid a within-patient bias. Histograms show the distribution of samples and groups on PC1 and PC2. This was repeated using only the first (up) or last (down) sample per patient. On the right are boxplots of PC1 (left) and PC2 (right), similar to Fig. 1D. *q<0.05, **q<0.01, ***q<0.001, Mann- Whitney test with Benjamini–Hochberg FDR correction.


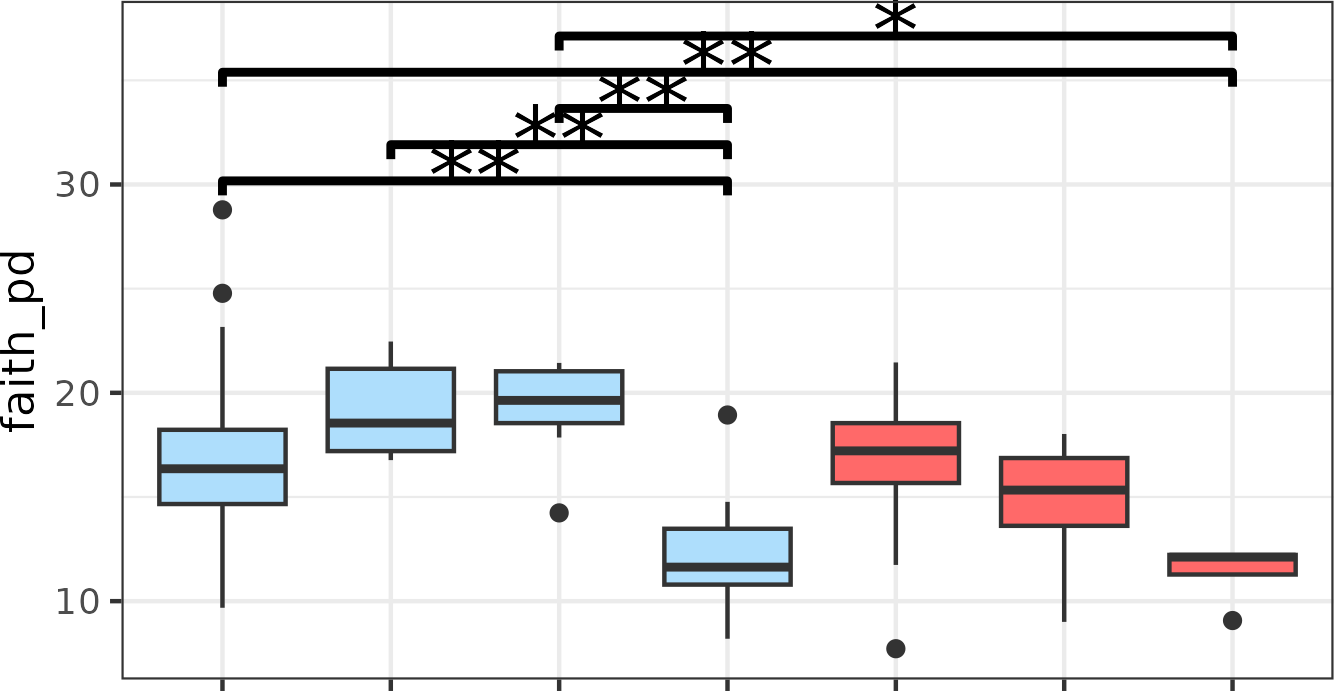
Antibiotics within 4W


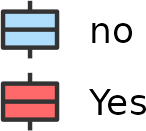


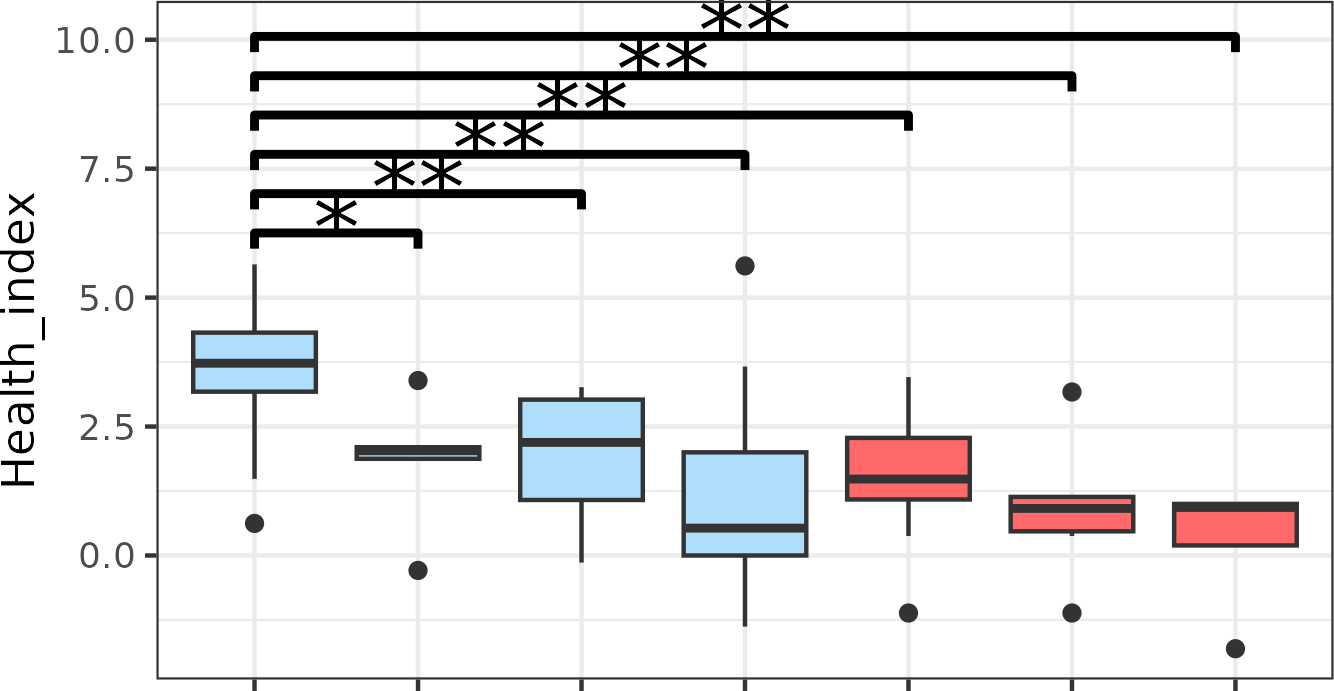
Antibiotics within 4W


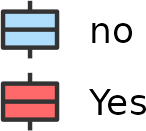


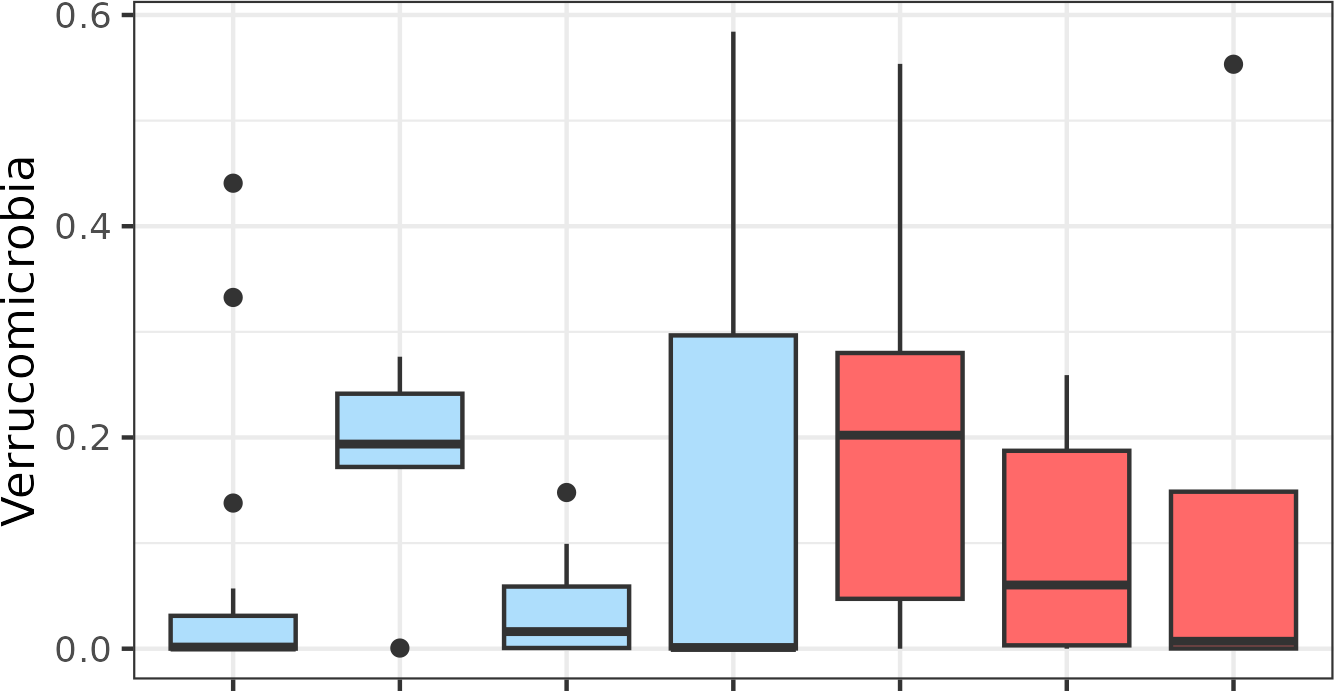
Antibiotics within 4W


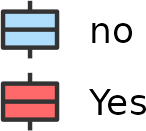


**Fig. S2. Stratifying by antibiotics exposure within 4 weeks and including one sample per subject.** Boxplots of alpha diversity values, using Faith’s phylogenetic diversity (upper), health index (middle), and *Verrucomicrobia* phylum RA (lower), stratified by group and antibiotics usage in the last four weeks. To avoid a within- subject bias within the sub-acute longitudinal samples, only the first and last samples are shown, separately. *q<0.05, **q<0.01, Mann-Whitney test with Benjamini– Hochberg FDR correction.


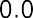

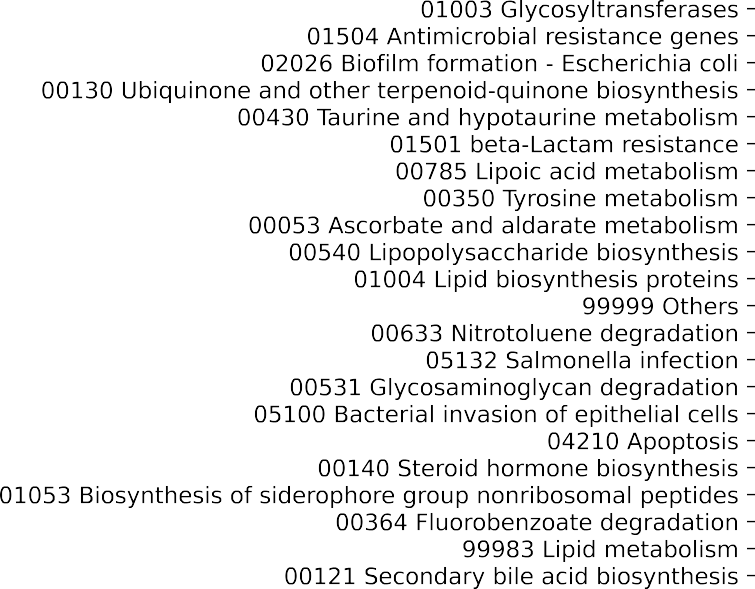

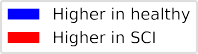


**Fig. S3. Enriched KEGG ontology functions associated with healthy and SCI patient- associated ASVs.** Level 3 KEGG ontology terms were predicted for the healthy and SCI patient-associated ASVs (the set of 54 and 115 ASVs that were higher and lower respectively between SCI and controls, detailed in Figure 3B and Table S3). KEGG ontology level functions significantly enriched in the healthy or SCI patient-associated ASVs were identified using a non-parametric rank-mean test with multiple hypothesis correction (dsFDR=0.1). Blue and Red bars denote level 3 KEGG ontology terms significantly enriched in the healthy and SCI patient-associated ASVs respectively.

1. ASVs higher in subjects with hard stool

(p=1E-8)


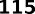


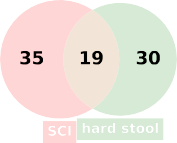


# C


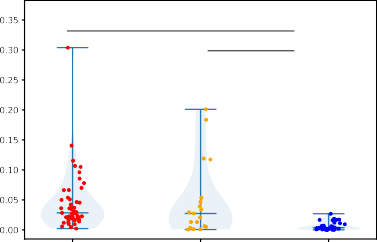


p=6E-11

p=5E-4

1. ASVs higher in subjects with normal

stool (p=5E-7)


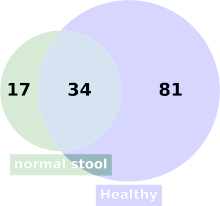

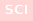


# D


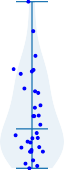

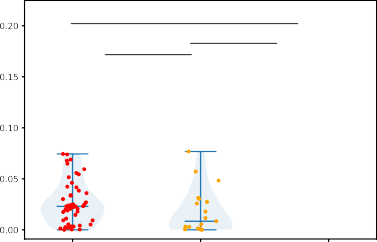


p=2.1-6

p=9.1E-2 p=5.4E-6

subacute chronic

Control

subacute chronic

Control

**Fig. S4. Similarities between bacteria associated with SCI, and stool consistency in the American Gut cohort. A**. Overlap between ASVs significantly higher in SCI in our study (red circle), ASVs significantly higher in healthy controls (blue circle), and ASVs significantly higher in American Gut participants with stool Bristol scores 1-2 compared (hard stool) to stool Bristol scores 3-4 (normal stool) (green circle, dbBact annotation 8447). **B.** Similar to Figure S4A (red and blue circles remained as 5A) but comparing to ASVs significantly higher in American Gut participants with stool Bristol scores 3-4 (normal stool) compared to stool Bristol scores 1-2 (hard stool) (green circle, dbBact annotation ID 8448). **C.** Per sample distribution of the weighted dbBact F-score for the American Gut annotations containing the term “hard stool” across the three cohort sub- groups (Distribution lines representing min median and max for each subgroup) showing that the bacterial composition of healthy controls from our study is less associated with hard stool ASVs as reported in the American Gut study (Horizontal bars denote significant differences between groups with mentioned p-values by nonparametric Man-Whitney tests). **D.** Similar to Figure S4C, but using F-Score for the American Gut annotations containing the term “normal stool”.
